# Retrospective T2 quantification from conventional weighted MRI of the prostate based on deep learning

**DOI:** 10.3389/fradi.2023.1223377

**Published:** 2023-10-11

**Authors:** Haoran Sun, Lixia Wang, Timothy Daskivich, Shihan Qiu, Fei Han, Alessandro D'Agnolo, Rola Saouaf, Anthony G. Christodoulou, Hyung Kim, Debiao Li, Yibin Xie

**Affiliations:** ^1^ Biomedical Imaging Research Institute, Cedars-Sinai Medical Center, Los Angeles, CA, United States; ^2^ Department of Bioengineering, University of California Los Angeles, Los Angeles, CA, United States; ^3^ Minimal Invasive Urology, Cedars-Sinai Medical Center, Los Angeles, CA, United States; ^4^ Imaging/Nuclear Medicine, Cedars-Sinai Medical Center, Los Angeles, CA, United States; ^5^ Imaging, Cedars-Sinai Medical Center, Los Angeles, CA, United States

**Keywords:** prostate cancer, deep learning, multiparametric MRI, quantitative imaging, T2 mapping, peripheral zone, active surveillance

## Abstract

**Purpose:**

To develop a deep learning-based method to retrospectively quantify T2 from conventional T1- and T2-weighted images.

**Methods:**

Twenty-five subjects were imaged using a multi-echo spin-echo sequence to estimate reference prostate T2 maps. Conventional T1- and T2-weighted images were acquired as the input images. A U-Net based neural network was developed to directly estimate T2 maps from the weighted images using a four-fold cross-validation training strategy. The structural similarity index (SSIM), peak signal-to-noise ratio (PSNR), mean percentage error (MPE), and Pearson correlation coefficient were calculated to evaluate the quality of network-estimated T2 maps. To explore the potential of this approach in clinical practice, a retrospective T2 quantification was performed on a high-risk prostate cancer cohort (Group 1) and a low-risk active surveillance cohort (Group 2). Tumor and non-tumor T2 values were evaluated by an experienced radiologist based on region of interest (ROI) analysis.

**Results:**

The T2 maps generated by the trained network were consistent with the corresponding reference. Prostate tissue structures and contrast were well preserved, with a PSNR of 26.41 ± 1.17 dB, an SSIM of 0.85 ± 0.02, and a Pearson correlation coefficient of 0.86. Quantitative ROI analyses performed on 38 prostate cancer patients revealed estimated T2 values of 80.4 ± 14.4 ms and 106.8 ± 16.3 ms for tumor and non-tumor regions, respectively. ROI measurements showed a significant difference between tumor and non-tumor regions of the estimated T2 maps (*P *< 0.001). In the two-timepoints active surveillance cohort, patients defined as progressors exhibited lower estimated T2 values of the tumor ROIs at the second time point compared to the first time point. Additionally, the T2 difference between two time points for progressors was significantly greater than that for non-progressors (*P* = 0.010).

**Conclusion:**

A deep learning method was developed to estimate prostate T2 maps retrospectively from clinically acquired T1- and T2-weighted images, which has the potential to improve prostate cancer diagnosis and characterization without requiring extra scans.

## 1. Introduction

Prostate cancer (PCa) is one of the most common cancer types in men. According to Global Cancer Statistics (1), PCa accounts for the second highest incidence of cancer among men and remains a leading cause of mortality. And the majority of new low-risk prostate cancer diagnoses will be managed with active surveillance (AS). In recent years, multiparametric MRI (mpMRI) has been recommended as a noninvasive imaging tool to improve the diagnostic pathway for PCa. mpMRI combining T2-weighted, diffusion-weighted imaging (DWI), and dynamic contrast-enhanced (DCE) imaging has shown excellent clinical value in cancer detection, biopsy targeting, risk stratification, staging, and treatment planning of PCa (2–4). Current clinical guidelines adopt mpMRI as the primary noninvasive diagnostic tool for PCa (3, 5). However, limitations of mpMRI exist in several aspects, including low sensitivity for low-grade cancer detection (6, 7); a false negative rate of 10%–20% for diagnosing high-grade tumors (8); and interobserver variability among readers of varying levels of experience (9, 10).

Recently, quantitative MRI has been shown to improve PCa diagnosis and characterization compared to standard mpMRI (11–14). In contrast to the qualitative weighted images included in mpMRI, quantitative maps are more objective representations of the intrinsic physical properties and have higher repeatability and reproducibility. This helps to reduce the variations from both the observers (inter- and intra-) and scanners (15, 16). With these advantages, the measurable differences in relaxation times between normal and tumor tissue have been shown to improve prostate lesion characterization (17–19). And T2 maps are especially helpful in differentiating cancer from normal prostate tissue and determining its aggressiveness (12, 13, 20, 21). More recently, Hepp et al. showed that T2 mapping has high diagnostic accuracy for differentiating between PCa and chronic prostatitis, comparable to the performance of ADC values (21). However, one main challenge for quantitative MRI is the limited clinical availability, since acquiring quantitative maps requires additional pulse sequences that are either time-consuming (traditional protocols) or not widely available [advanced multiparametric mapping techniques, for example, MR fingerprinting (11)].

Deep learning-based methods are increasingly used in MR image synthesis, including as a translation approach between qualitative weighted images and quantitative maps (22). Several studies in brain have shown the potential of convolutional neural networks (CNN) in quantitative MRI estimation from conventional weighted images. Wu et al. (23) used self-attention deep convolutional neural networks to estimate T1, proton density, and B1 maps from T1-weighted images. Moya-Sáez et al. (24) and Qiu et al. (25) used CNN approaches to compute quantitative T1 and T2 maps in the brain using clinical contrast-weighted images as inputs. These deep learning approaches do not require additional quantitative MRI scans and have the potential to improve the availability of quantitative information.

In this study, we developed a deep learning-based method to directly estimate T2 maps of the prostate from clinically acquired T1- and T2-weighted images. Because of the potential motion-induced position mismatch between different image sets in prostate imaging, various specific preprocessing steps, such as mask generation and deformable registration were applied to address this challenge. The proposed network was trained and validated using *in vivo* prostate MR scans from both PCa patients and healthy volunteers, followed by a retrospective T2 quantification on two patient groups, prostate cancer cohort and AS cohort, to further explore the potential of this approach in clinical practice.

## 2. Materials and methods

### 2.1. Study subjects and dataset

In vivo studies were approved by the institutional review board of Cedars-Sinai Medical Center. Informed consent was obtained from all study subjects before enrollment. Three groups of subjects were scanned on a 3 T clinical scanner (Biograph mMR; Siemens Healthineers, Erlangen, Germany) as listed below. Detailed MRI parameters of implemented protocols are listed in Table 1.

**Table 1:** Protocol parameters of the acquisitions in all groups included in this study (group 1 and 2).

	Group 1a				
	Group 1b and 2				
	T1w (FLASH)	T2w (TSE)	DWI (EP)	DCE (GRE)	T2 map (multi-echo spin-echo)
TE (ms)	2.03	108	95	1.07	10.5, 21.0, 31.5, 42.0, 52.5, 63.0, 73.5, 84.0
TR (ms)	250	3,000	6,500	3.02	4,980
α (°)	48.0	140	90	10	180
# Slices	40 (axial)	40 (axial)	29 (axial)	31 (axial)	30 (axial)
Thickness (mm)	6	6	3	3	3
Resolution (mm²)	1.125 × 1.125	1.125 × 1.125	0.781 × 0.781	1.250 × 1.250	1.172 × 1.172
FOV (mm^2^)	225 × 360	292 × 360	200 × 200	160 × 160	300 × 300
Temporal Res (s)	–	–	–	20	
b-value (s/mm^2^)	–	–	50, 800, 1,400	–	
Scan time (min)	0.5	1.0	6.4	8.2	∼ 12.0

Conventional multiparametric MRI was acquired in all groups. Additional T1 mapping reference was acquired in group 1a.

#### 2.1.1. Group 1a (w/reference T2)

It contains twenty-five subjects, including seventeen confirmed PCa patients and eight healthy volunteers. For each subject, conventional weighted images were acquired, including T1-, T2-weighted images, DWI and DCE. Reference T2 maps were acquired using a multi-echo spin-echo sequence and then generated by exponential fitting without the image of the first echo.

#### 2.1.2. Group 1b (w/o reference T2)

It contains twenty-four confirmed PCa patients scanned on the same scanner with the identical protocol settings as Group 1a, except without reference T2 map.

#### 2.1.3. Group 2 (as cohort)

**Figure 1:**
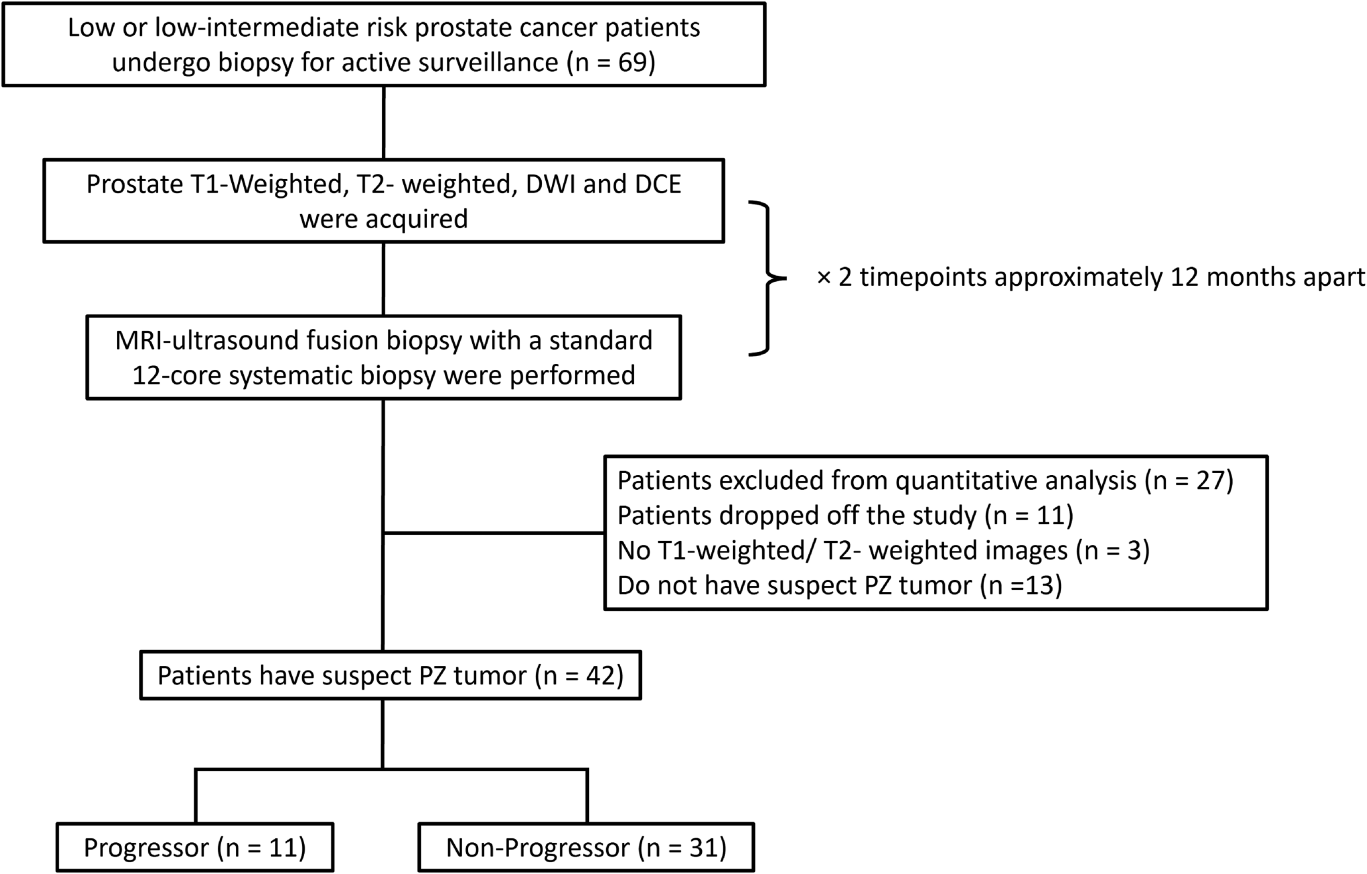
The inclusion workflow of the group 2 dataset (AS cohort). Patients who were identified by the radiologist to have peripheral zone tumor and meet the progressor criteria were included in the progressor set (*n* = 11), whereas others were included in the non-progressor set (*n* = 31). PZ = peripheral zone.

It consists of forty-two patients with low or low-intermediate risk prostate cancer undergoing AS. At two different time points, two conventional multiparametric MRIs were approximately 12 months apart (10.7 ± 2.2 mo). Each MRI was followed by a prostate biopsy conducted between September 2017 and December 2022. Progression criteria were defined based on biopsies as adverse histology presence (Gleason score ≥7) or an increase of 3 or more positive cores examined. Lesions with histopathologic diagnoses other than suspicious peripheral zone lesions were excluded from quantitative ROI analysis (Figure 1).

### 2.2. Data preprocessing

The workflow of preprocessing for Group 1a includes two parts, conventional weighted image preprocessing and T2 map image preprocessing. For the conventional weighted images, both T1- and T2-weighted images were first resampled to match the spatial resolution of T2 maps. Then the weighted images and T2 map images were cropped to a smaller field of view to avoid artifacts caused by the saturation band used during T2-weighted scanning. All conventional weighted images were deformable-registered to one echo time image from the T2 mapping sequence (TE = 84.0 ms, the closest TE to the conventional T2-weighted images), taking advantage of their similar contrast. For Group 1b and Group 2 datasets, spatial resolution correction was first implemented, then co-registration was only implemented between the weighted images. After cropping all the images to the same size to cut off the zero-value pixels caused by the alignment transform using ANTsPy (26), 24 slices were available for each subject.

A morphological image processing algorithm was developed to generate the bladder mask based on the T1-weighted image, followed by manual correction. For each subject, conventional weighted images were normalized by the mean plus three times the standard deviation of the whole 3D image volume, excluding the bladder pixels. T2 maps were scaled by 400 ms, since the range of 0–400 ms covers most pelvis tissue. Extreme value masks (T2 value larger than 400 ms) were generated in order to avoid spurious high-intensity pixels in the T2 maps, which would otherwise affect both the network training and evaluation.

### 2.3. Deep learning network training

**Figure 2:**
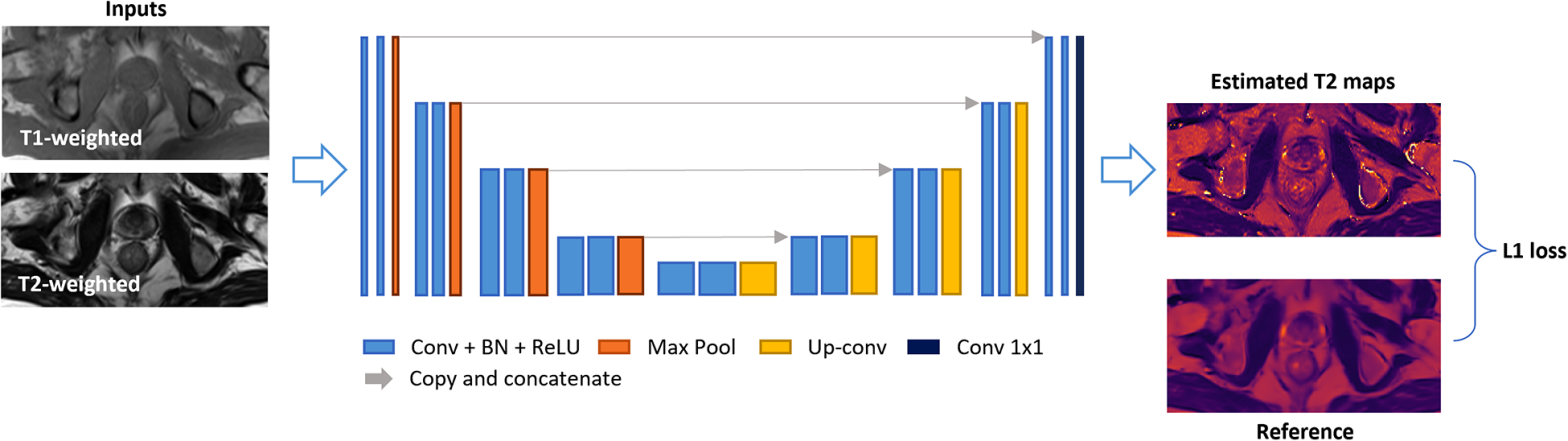
Deep-learning network structure. A 2D U-Net-based architecture consisted of four down-sampling steps and four up-sampling steps was implemented. Each encoder stage was followed by 2 × 2 max-pooling for down-sampling, and each decoder stage was followed by 2 × 2 up-sampling convolutional layers. Every stage incorporated two series of 3 × 3 2D convolutions, batch normalization, and rectified linear units (ReLU). Input images included T1- and T2-weighted images concatenated as two channels. The output image was an estimated T2 map. An L1 loss function was used.

A 2D U-Net-based network was trained to estimate T2 maps from T1- and T2-weighted images, which were concatenated as 2-channel inputs. The detailed network architecture is illustrated in Figure 2. The model consisted of a four-step contraction path that encoded high-resolution data into low-resolution representations and a four-step expansion path that decoded such encoded representations back to high-resolution images. Both the encoder and decoder parts were modified based on the U-Net structure (27), where each stage consisted of two series of 3 × 3 2D convolutions, batch normalization, and rectified linear units (ReLU). In the encoder part, each stage was followed by a 2 × 2 max-pooling for down-sampling, while for the decoder part, four 2 × 2 up-sampling layers converted low-resolution representation back to high resolution.

A four-fold cross-validation was implemented on Group 1a dataset (twenty-five subjects) with a training:validation:testing split of 19:2:4. This strategy covered all the PCa subjects in testing and the inference results would be used for evaluation. An L1 loss function between the network outputs and the reference maps was minimized with the ADAM optimizer (28), as L1 loss is more robust to potential misregistration. The model was implemented using PyTorch with CUDA-11.6, NVIDIA RTX 1080-TI GPU.

### 2.4. Evaluation

#### 2.4.1. Global analysis

Image-based error evaluation was performed on the inference results from the seventeen suspected PCa patients in Group 1a dataset, which have acquired T2 maps as references. Mean percentage error (MPE), peak signal-to-noise ratio (PSNR), and structural similarity index (SSIM) were calculated to assess the error at both the voxel level and structure level. The Pearson coefficient was also calculated to assess the correlation between the estimated T2 values and the reference T2 values. All the metrics were evaluated slice by slice while masking out the bladder region and the extreme value pixels.

To assess the effectiveness of the designed processing pipeline, the metrics were also calculated using similar deep learning approaches but without deformable registration and/or bladder masking. The results were compared with the proposed approach.

#### 2.4.2. Regional analysis

For Group 1a, an experienced radiologist provided both tumor and non-tumor ROI labeling for the T2-weighted image and T2 map ground truth respectively using ITK-SNAP (www.itksnap.org) (27). Tissue segmentation was informed by the standard clinical reading workup based on mpMRI and PSMA-PET (when available). The independent ROI labels drawn on each acquisition can represent the tumor and non-tumor regions more accurately since the deformation of the prostate in different image sequences cannot be avoided or eliminated. Same tumor and non-tumor ROIs labeling have been performed on the T2-weighted images of Group 1b dataset; only tumor ROIs have been labeled on the T2-weighted images of Group 2 dataset.

For the fourteen suspected PCa patients in Group 1a dataset, mean T2 values were calculated in the ROI labels for each tumor and non-tumor region. Comparisons were performed between the estimated T2 map and the T2 map ground truth. Paired *t*-tests with a significance level of *P* = 0.05 were performed to test whether there are significant differences between the estimated T2 values and the reference T2 values of both tumor and non-tumor regions. Also, paired *t*-tests were conducted between the tumor and non-tumor regions to evidence that the network can improve PCa diagnosis and characterization potentially. To further test the network performance in T2 quantification of PCa (focusing on peripheral zone lesion), Group 1b dataset without corresponding T2 maps were also input to the trained network. And the estimated T2 maps were used to calculate the mean T2 value of the tumor and non-tumor ROIs then followed by a paired *t*-test with a significance level of *P* = 0.05. Statistical ROI analysis was also performed on the estimated T2 maps of the thirty-eight cases of Group 1 in total.

For the Group 2 dataset, T1-weighted and T2-weighted images from two time points were used to generate estimated T2 map using the same trained network. Quantitative analysis was conducted by comparing the mean estimated T2 values of the same tumor ROIs between the two time points. The differences between the two time points were denoted as deltaT2 (timepoint 2 min timepoint 1). Paired *t*-tests were performed separately for the progressor and non-progressor sets between timepoint 1 and timepoint 2. Un-paired *t*-tests were also carried out on the same time points for progressor and non-progressor sets, as well as on the deltaT2 values for both sets.

## 3. Results

### 3.1. Global analysis

**Figure 3:**
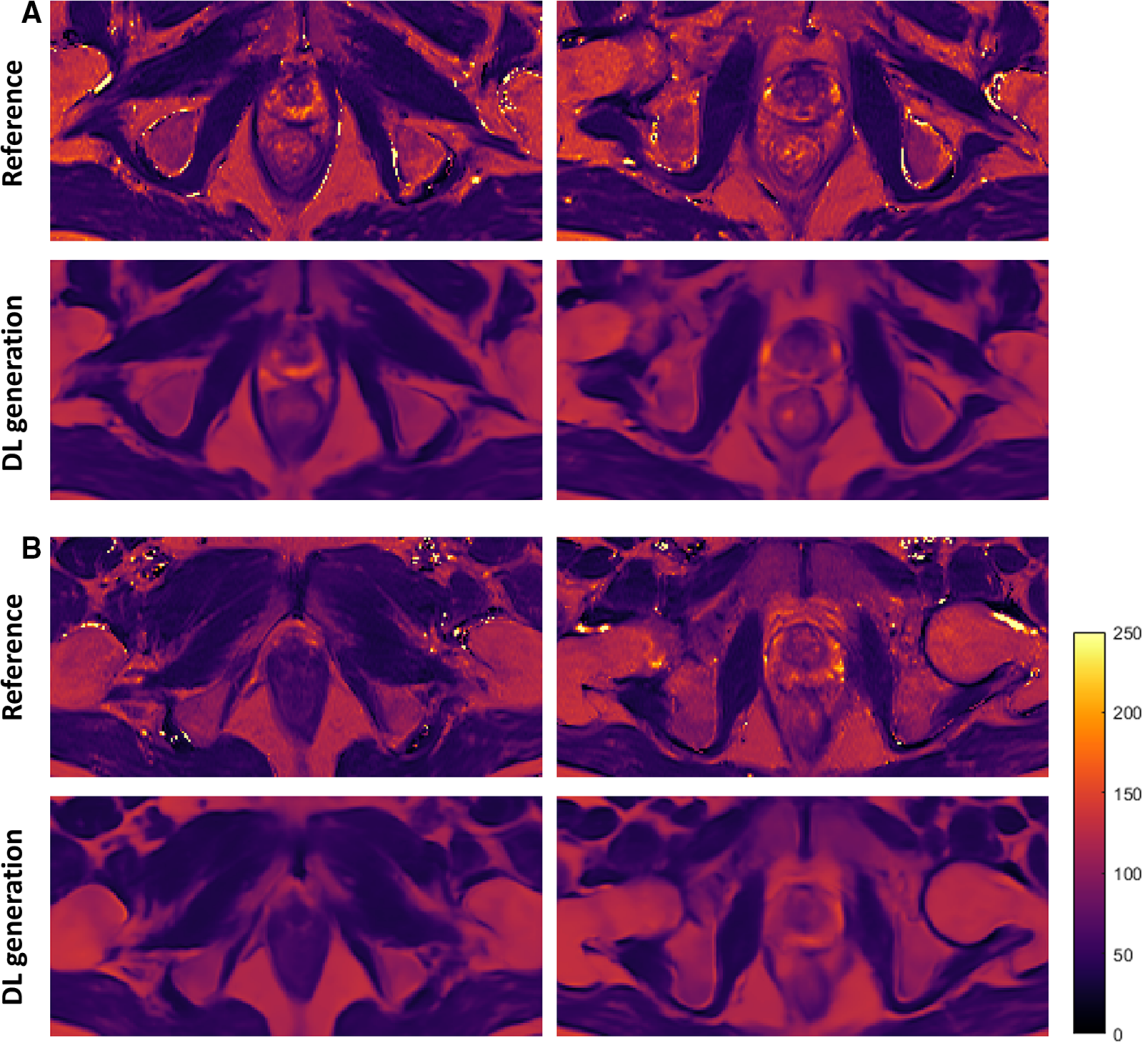
Representative slices of the estimated T2 maps and the corresponding ground truth from two PCa patients (**A,B**). The first and third rows are the T2 references, and the second and fourth rows are the estimated T2 maps generated by the deep learning network. The two columns represent two slices which include prostate glands. DL, deep learning.

The resulting estimated T2 maps are visually similar to the ground truth. Figure 3 shows four representative slices from two PCa patients. Tissue structures and contrast are well preserved with high similarity to the T2 map ground truth. Also, the results generated by the trained network are smoother and do not contain pixels that have extremely high intensity.

Table 2 shows the quantitative analysis results of the proposed method as well as the alternative approaches where deformable registration and/or bladder masking were not included. Compared with the first row which shows the performance of the basic U-Net model, the second and third rows show improved performance of the network after adding deformable registration or bladder masking approach.

**Table 2:** Quantitative analysis results of the estimated T2 map of the seventeen PCa patients in group 1a on the whole image level with different approaches.

Method	PSNR	SSIM	MPE (%)	Pearson correlation coefficient
U-Net	24.92 ± 2.00	0.81 ± 0.04	22.34 ± 4.96	0.77
U-Net + Deformable registration	25.77 ± 1.73	0.84 ± 0.03	18.55 ± 2.55	0.82
U-Net + Bladder masking	25.25 ± 1.39	0.81 ± 0.05	21.09 ± 4.58	0.81
U-Net + Deformable registration + Bladder masking	26.41 ± 1.17	0.85 ± 0.02	17.78 ± 1.81	0.86

The first row shows the performance of the basic U-net model. The second and third row show the quantitative analysis results with deformable registration or bladder masking in the prostate T2 estimation process. The last row shows the finial performance of the proposed method.

The quantitative analysis results of the proposed approach are listed in the last row. Compared with the T2 map references, the estimated T2 maps yielded a PSNR of 26.41 ± 1.17 dB, an SSIM of 0.85 ± 0.02, and an MPE of 17.78%. In addition, the correlation analysis showed a strong relationship between the estimated maps and the corresponding ground truth, with a Pearson correlation coefficient of 0.86.

### 3.2. Regional analysis

**Figure 4:**
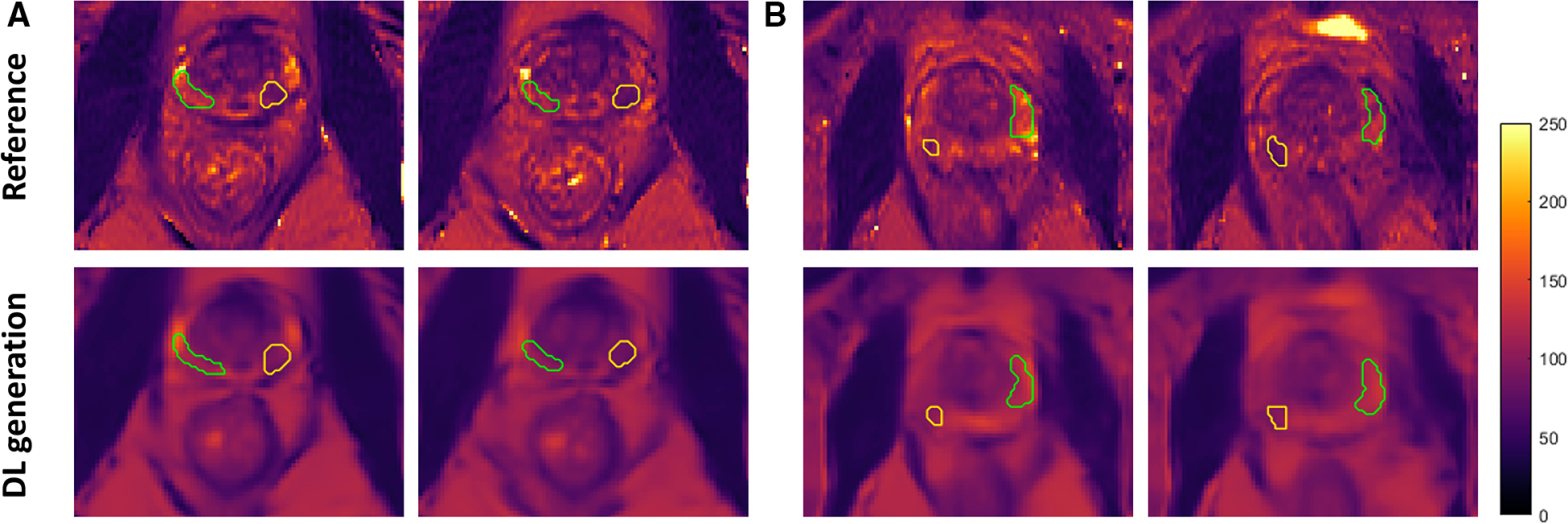
Zoomed-in view of representative slices of the estimated T2 maps and the corresponding ground truth from two PCa patients (**A,B**) with ROI labeled. Both tumor (yellow) and non-tumor (green) regions were outlined on the peripheral zone of the prostate gland. DL, deep learning.

Among the PCa patients in the testing set of the Group 1a dataset which has corresponding T2 maps as references, fourteen of them have lesions located in the peripheral zone of the prostate included in the ROI analysis. Figure 4 shows zoomed-in view of the prostate gland of two representative PCa patients, with the tumor region outlined yellow while the non-tumor region outlined in green. The different signal intensity of the tumor and non-tumor region was accurately estimated by the network consistent with the trend reported in literature (14, 17, 20, 29).

**Figure 5:**
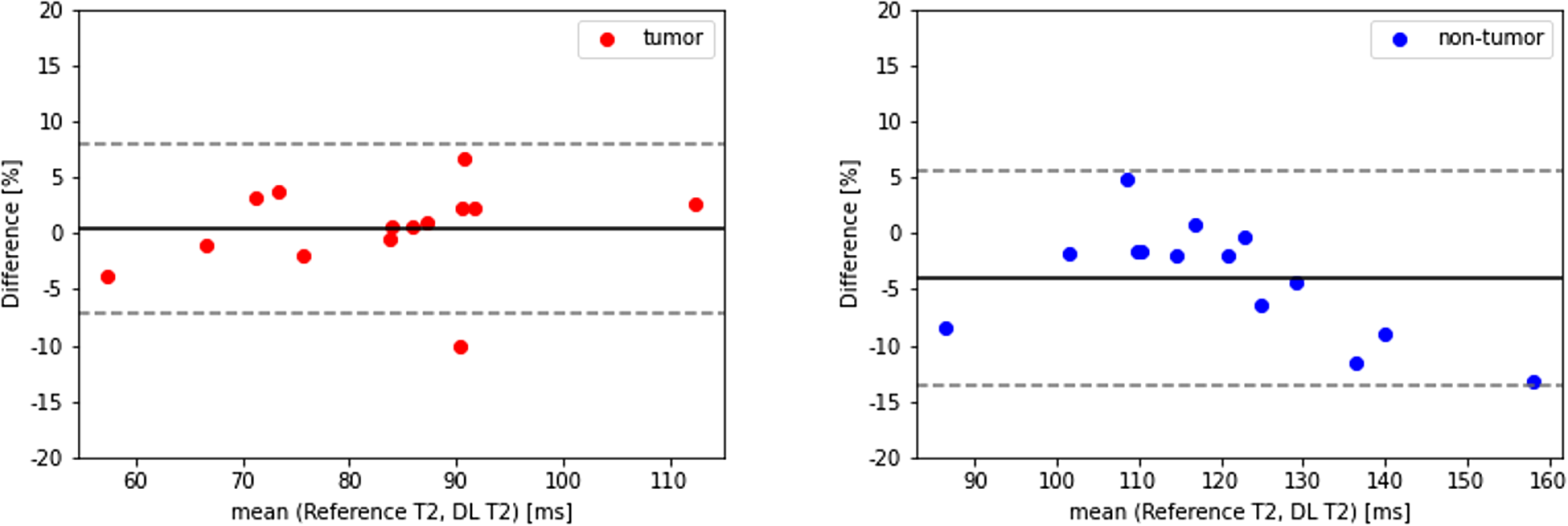
Bland-Altman plots of tumor (left, red) and non-tumor (right, blue) regions in 14 PCa patients (group 1a). DL, deep learning.

Table 3 shows the T2 ROI measurements for the tumor and non-tumor regions of thirty-eight PCa patients. For Group 1a, the estimated T2 value of tumor region had a mean of 83.7 ± 16.5 ms, which was not significantly different from the reference T2 value of 82.1 ± 13.5 ms, while the estimated T2 value of non-tumor region was 111.9 ± 14.2 ms, a 16 ms underestimation (*P *= 0.045) compared to the reference 128.1 ± 23.9 ms. In addition, a clear pattern emerged that T2 values of the tumor region were lower than the non-tumor region, which was consistent with the T2 map ground truth. Both the estimated T2 values and the reference showed a significant difference between tumor and non-tumor. Figure 5 shows the Bland-Altman plots for the T2 values of tumor and non-tumor regions. The mean difference was smaller than ±1%, and the limits of agreement were within ±10% for the tumor regions. For the non-tumor regions, the mean difference was smaller than ±5%, and the limits of agreement were around +5% and −15%.

**Table 3:** T2 ROI measurements on tumor and non-tumor regions in thirty-eight PCa patients (group 1).

Group 1a (*N* = 14)		DL estimation	Reference	*P* value
T2 (ms)	Non-Tumor region	111.9 ± 14.2	128.1 ± 23.9	0.045*
	Tumor region	83.7 ± 16.5	82.1 ± 13.5	0.788
	*P* value	<0.001***	<0.001***	
Group 1b (*N* = 24)				
T2 (ms)	Non-Tumor region	103.8 ± 16.5		
	Tumor region	78.4 ± 12.6		
	*P* value	<0.001***		
Group 1 all (*N* = 38)				
T2 (ms)	Non-Tumor region	106.8 ± 16.3		
	Tumor region	80.4 ± 14.4		
	*P* value	<0.001***		

DL, deep learning.

Two series of paired *t*-tests were included in the analysis: one is between deep learning estimation and ground truth (only on Group 1a), and the other is between tumor and non-tumor regions with the significance level of *P < 0.05; ***P < 0.001.

To further evaluate the trained network, ROI analysis was also performed on the estimated T2 maps of Group 1b dataset. As shown in Table 3, a significant difference between the tumor and non-tumor regions was observed from the twenty-four estimated T2 maps, with a mean T2 value of 78.4 ± 12.6 ms of the tumor regions and a mean T2 value of 103.8 ± 16.5 ms of the non-tumor regions, which consistent with the results from Group 1a. Quantitative ROI analyses performed on the whole Group 1 dataset showed an estimated T2 values of 80.4 ± 14.4 ms and 106.8 ± 16.3 ms for tumor and non-tumor regions with a significant difference level of *P *< 0.001.

**Figure 6:**
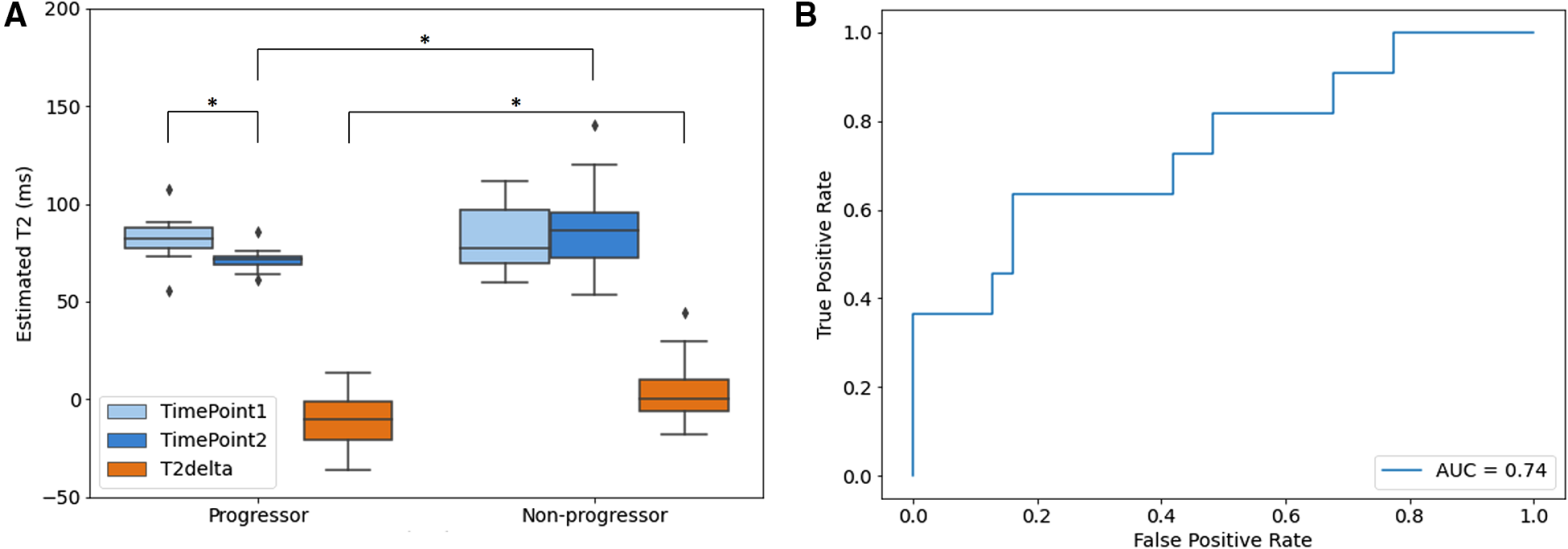
Quantitative analysis results of aS dataset (group 2). (**A**) Box and whisker plot of the mean estimated T2 value of ROIs. Timepoint 1 (light blue), timepoint 2 (blue) and the corresponding T2 delta (orange) of progressor and non-progressor were shown separately with significance level of *P < 0.05; (**B**) ROC curve of the delta T2 value between timepoint 1 and timepoint 2. The boxes represent the interquartile range between 25–75th percentiles. The lines within boxes represent median value. The whiskers represent measurements 1.5 times interquartile range. The points represent outliers beyond 1.5 times the interquartile ranges.

For the Group 2 dataset, the AS data cohort, the ROI analysis results are presented in Figure 6. The estimated T2 maps generated by the proposed method were measured for both progressor and non-progressor tumor ROIs. The estimated tumor T2 values in the progressors and non-progressors were 82.1 ± 12.1 ms and 82.6 ± 15.8 ms, respectively, for timepoint 1; and 71.4 ± 6.1 ms and 85.9 ± 19.2 ms for timepoint 2. The differences in tumor T2 between the two timepoints, calculated as timepoint 2 min timepoint1, were denoted as T2delta. The mean estimated T2 delta for the progressor and non-progressor were −10.7 ± 14.2 ms and 3.4 ± 14.5 ms, respectively. The distributions of timepoint 1, timepoint 2 and the corresponding T2 delta were depicted as box-and-whisker plots in Figure 6A. For the progressor set, timepoint 2 showed a relatively lower T2 value compared to timepoint 1, while the T2 values of the non-progressor set overlapped between the two timepoints. The 25th to 75th percentiles of T2delta for the progressor were distributed below zero, whereas those for the non-progressor were centered around zero. Paired *t*-tests only revealed a significant difference between the two time points for the progressor (*P *= 0.039). Un-paired *t*-tests showed significant differences between the two groups for timepoint 2 (*P *= 0.021) and for the T2deltas of progressors and non-progressors (*P *= 0.010). No significant difference was observed between the two groups at timepoint 1. The ROC curve of using T2delta values for classifying progressors from non-progressors shows an AUC of 0.74.

## 4. Discussion

In this work, a deep learning-based approach was developed to estimate T2 values from clinical T1- and T2-weighted images. The estimated T2 maps showed a similar image contrast compared with the T2 map ground truth while preserving the pelvic tissue structure details with high agreement to the reference. The quantitative metrics demonstrated the feasibility of the proposed method in estimating T2 maps. The ROI analysis results provided further evidence of the effectiveness of the proposed approach in T2 quantification, as well as the differentiation ability between tumor and non-tumor regions also the progressor and non-progressor.

This is the first deep learning-based prostate T2 estimation based on conventional contrast-weighted images. The information from multiple contrast-weighted images is merged with a neural network, where the prostate MRI data presents specific challenges because of the deformation between different acquisitions. For prostate imaging, bladder filling makes the deformation problem complex and unavoidable. To address the issues, our developed pipeline includes optimized preprocessing steps. Deformable registration between weighted images and T2 references contributed to establishing spatial correspondences between the difference image acquisitions. Special care was also taken on bladder regions, in the proposed processing, masking out the irrelevant information carried by the bladder area improved both the normalization of input weighted image and the training of the network.

For the ROI analysis of tumor and non-tumor regions, the estimated T2 maps showed lower T2 values in tumor regions than in non-tumor regions in PCa patients, which is a trend consistent with the findings reported in recent clinical papers using dedicated T2 mapping sequences (12, 29). No significant difference in tumor T2 was observed between the proposed method and the reference method. A small bias (16 ms) was observed in the non-tumor regions between the estimated T2 values and the reference. This may be related to the mismatch in some cases with extreme values. For these cases, the reference T2 maps showed extremely high T2 values (e.g., 250 ms) in the non-tumor region, while the estimated T2 values were stable and lower than the reference ones. This mismatch may be due to the low occurrence of the pixels with extreme values in the training data. Enlarging the dataset to include a wider range of weighted images with corresponding references may increase the dynamic range of the output.

The majority of men with low-risk prostate cancer are managed through AS, and mpMRI has been explored for identifying and monitoring AS patients (30–32). Previous investigations have shown high diagnostic accuracy of T2 relaxometry in predicting prostate cancer aggressiveness, comparable to the performance of ADC values (22). In this study, quantitative analyses were performed on AS patient cohort. For the progressor set, the estimated T2 values of the tumor decreased from timepoint 1 to timepoint 2, while no significant difference was observed in the T2 values of the tumor between the two time points. The T2 delta between the two time points for progressors was significantly different from that for non-progressors. The estimated T2 value holds the potential to increase the predictive value of mpMRI for progressive prostate cancer.

In this work, only prostate T2 was retrospectively quantified, as its value has been extensively investigated for prostate disease diagnosis. T1-weighted imaging, on the other hand, has not been used routinely for clinical decision-making. If needed, the proposed approach may be adapted to quantify T1 in the future.

The current study has several limitations. There was a slight level of visible blurriness in the estimated T2 maps compared with the reference T2 maps. One possible reason is that the input T1 and T2 weighted images have lower resolution than the T2 maps. Despite the use of bladder masking and deformable registration, there is still residual misregistration between the input images, which could contribute to the reduction in image sharpness. Registration methods that are more robust than the current pairwise deformable registration performed by ANTs will be further explored. Also, the dataset used in this study was of limited size: there were only 600 slices in total available from eight healthy volunteers and seventeen suspected PCa patients. Among the seventeen PCa patients, only fourteen of them had peripheral zone lesions, whereas lesions of the other three cases are located at the transition zone. In the future, a larger data set with references should be included to improve the supervised network performance. Moreover, an inclusion of a greater number of patients with transition zone tumors would advance the exploration of potential application. In addition, further tuning of the implemented CNN, or experimenting with other more sophisticated networks [for example, generative adversarial networks (33) and Transformers (34)], has the potential to further improve T2 mapping performance. Moreover, this is a retrospective study, the imaging protocols were set up with specific spatial resolution and echo times on one 3 T scanner. The conventional weighted images used to develop the network have a limited spatial resolution with a larger FOV compared with the current international guidelines (35). Future work should include data from multi-center, and different scanners and protocols to test and improve the generalizability of the proposed method.

## 5. Conclusion

Quantitative T2 maps of the prostate can be estimated from clinical contrast-weighted images using a deep learning neuro network with a high level of agreement with prospectively acquired reference. Preliminary studies in prostate cancer patients showed a significant difference in estimated T2 values between tumor and non-tumor regions using the estimated T2 maps. In patients on active surveillance, estimated T2 difference at two points showed lower value in progressors than the non-progressors. Upon further validation, this method has the potential to retrospectively derive T2 values from standard clinical MRI for more accurate PCa diagnosis and characterization.

## Data Availability

The original contributions presented in the study are included in the article, further inquiries can be directed to the corresponding authors.
